# Dietary nucleotide improves markers of immune response to strenuous exercise under a cold environment

**DOI:** 10.1186/1550-2783-10-20

**Published:** 2013-04-08

**Authors:** Joan Riera, Victoria Pons, Daniel Martinez-Puig, Carlos Chetrit, Josep A Tur, Antoni Pons, Franchek Drobnic

**Affiliations:** 1 Sports Physiology Department, Centre d’Alt Rendiment (CAR-GIRSANE), Av. Alcalde Barnils 3-5, Sant Cugat del Vallés, Barcelona, 08174, Spain; 2 Bioiberica S.A., Ctra. N-II km 680.6, Palafolls, Barcelona, 08389, Spain; 3 Laboratory of Physical Activity Science, Universitat de les lles Balears, Ctra. Valldemossa Km 7.5, Palma de Mallorca, E07122, Spain

**Keywords:** Exercise, Dietary nucleotides, Lymphocyte proliferation

## Abstract

**Background:**

Strenuous exercise has been classically associated to immune-suppression and consequently to an increased risk of infections, especially at the upper respiratory tract. The administration of dietary nucleotides has been demonstrated useful to maintain the immune function in situations of stress and thus could be an appropriate strategy to counteract the decline of the immune function associated to strenuous exercise. The aim of the present study was to asses the impact of a specific nucleotide formulation (Inmunactive®) on the markers of immune function of athletes after a heavy exercise bout under cold conditions.

**Methods:**

Twenty elite male taekwondo athletes were randomly divided into two groups of 10 subjects that were supplemented with placebo (P) or Inmunactive (I) at 480 mg/day during 30 days. At baseline (day 0) and after 4 wk of supplementation (day 30) each subject undertook an exhaustion exercise test using a cycloergometer. Skin temperature, core temperature, heart rate, lactate concentration and rating of perceived exertion (RPE) were recorded during the test. Blood and saliva samples were obtained before and after each exercise test for determination of blood cell concentrations, PHA-stimulated lymphocyte proliferation (PHA-LP) and salivary immunoglobulin A (SIgA).

**Results:**

Exercise tests induced neutrophilia and reduction in lymphocyte blood counts on day 0 and on day 30 in both groups. However, the I group exhibited a faster recovery from the lymphopenic response than the P group, so that lymphocyte levels were higher after 150 min (P < 0.0028). Furthermore, the lymphoproliferative response was modulated by nucleotide supplementation, since it was higher in the I group on day 30 despite an almost significant (P < 0.06) exercise-evoked decrease at baseline.

**Conclusions:**

These findings suggest that supplementation with a nucleotide-based product for 4 weeks could counteract the impairment of immune function after heavy exercise.

## Background

Epidemiologic studies show that, while moderate activity may enhance immune function above sedentary levels, acute bouts of prolonged high-intensity exercise impair immune function and are a predisposing factor to upper respiratory tract infections (URTI) [1-3]. Many studies have reported that some aspects of immune function, such as lymphocyte proliferation, or of secretory immunoglobulin A (IgA) concentrations in mucosal surfaces, are temporarily impaired after acute bouts of prolonged, continuous heavy exercise [1,4-7]. The elite athletes training requires repeated bouts of strenuous exercise in order to compete at the highest levels. Susceptibility to minor infections as a result of intensive endurance training is obviously a concern for athletes, as it is generally recognized that those minor infections result in a drop in exercise performance, interfere with the training program [8], and have been associated with the development of persistent fatigue [9]. Immune impairment has been associated to increased levels of stress hormones during exercise resulting in the entry into the circulation of less mature leukocytes from the bone marrow [3]. During exercise athletes are exposed to multiple stressors such as physical, psychological and environmental. Exposure to a cold environment affects the immune function, specially the lymphoproliferative responses [10]. Consequently, it has been demonstrated that vigorous exercise in cold temperatures is associated to increased susceptibility to URTI [11,12] even above what is observed with physical exercise alone [13].

Nucleotides are low molecular weight intracellular compounds, which play key role in nearly all biochemical processes [14]. As nucleotides can be synthesized endogenously they are not essential nutrients. However, under situations of stress, dietary nucleotides have been reported to have beneficial effects upon the immune system [14,15]. Although the molecular mechanisms by which dietary nucleotides modulate the immune system are practically unknown, it has been demonstrated that nucleotides influence lymphocyte maturation, activation and proliferation [16-18]. Likewise, they affect the lymphocyte subset populations [19,20], macrophage phagocytosis [17], immunoglobulin production [18,21], and delayed hypersensitivity as well as allograft and tumour responses [15,17]. Consequently, in several studies nucleotides supplementation has been shown to reverse the immune suppression associated to stress situations [22,23]. However, data available on endurance exercise trials is scarce. In controlled studies, it has been demonstrated that dietary nucleotides attenuates the fall of salivary IgA (SIgA) [24], the increase in salivary cortisol [25] and the decrease in the lymphocyte proliferative response [26] after the strenuous exercise. Nevertheless, aside from this study, there is no data available from prospective, double-blind, placebo-controlled studies, on the effects of nucleotide supplementation on the markers of immune response after strenuous exercise in a cold environment.

The aim of the present study was to test the impact of a specific nucleotide formulation (Inmunactive®, Bioiberica, Spain) on the immune function of athletes after a heavy exercise bout in cold conditions.

## Methods

### Subjects

Twenty elite male taekwondo players were recruited at the Centre d’Alt Rendiment (CAR) St. Cugat to participate in this study. Before being accepted to participate in the investigation, each subject performed a complete medical examination that included a medical history and resting ECG to screen for any medical problem that would contraindicate their participation in the study. The subject’s general physical characteristics were: 21.4 ± 6.3 years, 178.1 ± 8.5 cm, 73.86 ± 12.6 kg, 12.53 ± 3.2% percent body fat and 46.59 ± 5.7 ml · kg^-1^ · min^-1^ maximal oxygen uptake (VO_2max_).

This study was conducted according to the guidelines of the Declaration of Helsinki for Research on Human Subjects 1989 and was approved by the local Ethics Committee of the Consell Català de l’Esport (Generalitat de Catalunya).

### Research design

Two weeks before the first test, all the subjects performed a cycling maximal incremental test to determine their VO_2max_. Oxygen consumption was measured using a computerized metabolic cart (Master Screen CPX, Erich Jaeger, Wuerzburg, Germany), and the corresponding Watts at 60% (W_1_) 70% (W_2_) and 90% (W_3_) of VO_2max_ were calculated by linear interpolation. For the exercise test, subjects reported to the CAR laboratory at 8 a.m. after an overnight fast. Dry nude body weight was measured before and after the experiment following the subject had emptied the urinary bladder. The rate of dehydration was calculated by dry nude weight difference before and after testing. A saliva sample and a 8.5 mL blood sample were taken after a 10 min supine rest. Subjects were required to use the same clothes in both exercise sessions. The subjects entered into the climatic chamber, adjusted a cycle ergometer, placed the chest Hr transmitter and skin thermistors and undertook an exhaustion exercise test at work corresponding to W1 for 10 min, W_2_ for 20 min and W_3_ until fatigue in a climatic chamber adjusted at -3°C. Heart rate (Hr) was registered at rest and every 5 min during the exercise test using a chest Hr monitor (Polar Electro Inc, Kempele, Finland). Every 10 min a 20 μL blood sample was obtained from the ear lobule to analyze lactate concentration ([La]) (Dr. Lange® Berlin, Germany). Rate of perceived exhaustion (RPE) was recorded every 10 min during the test using the Borg scale [27]. Skin and core temperature (Tc) were continuously recorded during the exercise test. Tc was measured by intestinal pill system (Cor-Temp 2000®, HQInc, Palmetto, Florida, EEUU). The ingestible pill was swallowed approximately eight hours before the test to ensure passing into to gastrointestinal tract and T_c_ collected for analysis at rest and every 5 minutes during exercise, and after 5 minutes of recovery into the climatic chamber and was recorded using a telemetric sensor according the procedure described by Byrne [28]. Skin temperature was measured continuously with 4 skin thermistors (*CCI*® PT-100 W/0°C, Barcelona, Spain) placed in to the parasternal chest-side, mid arm, mid thigh and medial calf. The mean skin temperature (T_sk_) was calculated according to a Ramanathan formula [29] and collected for analysis at rest, every 5 minutes and after 5 minutes of recovery inside the climatic chamber. The average body temperature (T_m_) was calculated using the formula T_m_ = 0, 79 × T_c_ + 0, 21 × T_sk_[30]. Saliva samples were collected at 150 min after the end of the exercise test and blood samples were collected at 30 min, and 150 min for complete blood count (CBC) and at 24 h for the PHA-stimulated lymphocyte proliferation (PHA-LT) test.

### Dietary supplementation

Subjects agreed to avoid the use of large-dose vitamin/mineral supplements (>100% of recommended dietary allowances), herbs, and medications known to affect immune function during the entire 31-d study. Subjects recorded food intake in a 7-d food record before the first exercise test session and thorough the study. The food records were analyzed using a computerized dietary assessment program (ADN®, Barcelona, Spain). During orientation, a dietician instructed the subjects to follow a balanced diet and to no change habits during the study period.

After the first exercise test, each subject was randomly assigned to either the Inmunactive® (I) or placebo (P) group. Inmunactive® (Bioiberica, Barcelona, Spain) is a food supplement containing a mixture of free nucleotides (cytidine 5’-monophosphate, uridine 5’-monophosphate, adenosine 5’-monophosphate and guanosine 5’-monophosphate). The content of free nucleotides is 49.38 g/100 g. The commercial batch used for the study was D-01. The nucleotide content in the commercial batch used for the study (D-01) was confirmed analytically using a Waters 2695 (Milford, MA) HPLC system with a photodiode array extended λ detector Waters 2488.

Experimental products were provided under double-blind procedures. For blinding, a computer generated randomization number was assigned to unmarked boxes containing either Inmunactive® or placebo. The randomization code was maintained by the sponsor and concealed from the study site. Treatment allocation depended only on the time sequence in which patients entered the study, thus minimizing selection bias. All study-related case report forms recorded only the randomization number with unblinding only after the completion of the study or analyses.

Subjects in the nucleotide group (I) were treated with Inmunactive® at a dose of 972 mg · day^-1^ (2 capsules/day) for 30 days, while subjects in the placebo group (P) were treated during the same period with 2 capsules · day^-1^ containing excipient (microcrystalline cellulose).

Compliance was recorded during the study within the food records and monitored before the second exercise test.

Subjects agreed to maintain a steady training status which was recorded during the intervention period. After 30 days, subjects returned to the laboratory to undertake the second exercise test as described previously.

### Saliva analysis

Saliva production was stimulated by chewing a sterile cotton swab (Salivette; Sersted, Vümbrecht, Germany) during 60 seconds, and saliva was separated from the cotton by centrifugation at 2000 rpm × 5 minutes. Saliva samples were frozen at -80°C and stored until the end of the study period. SIgA concentration was analyzed using nephelometric quantification (BN™ II System, Siemens, Deerfield, IL, USA) according to the validated manufacturer protocol. Results were expressed in mg/L.

### Blood analysis

Blood samples (3.5 mL) were taken from the antecubital vein and collected in EDTA tubes. CBC was analyzed using the impedance system Abacus Junior® (Tecil, Barcelona, Spain).

### Phytohemagglutinin-stimulated lymphocyte proliferation

Blood samples (4 mL) were collected in heparinised tubes to analyze the lymphocyte proliferation rate. The mitogenic response of lymphocytes was determined in whole blood culture using phytohemaglutinin (PHA) at an optimal dose previously determined by titration experiments. Heparinized venous blood was diluted 1:10 with complete media consisting of RPMI-1640 supplemented with 5% heat-inactivated fetal bovine serum, penicillin, streptomycin, sodium pyruvate, L-glutamine, A2-mercaptoethanol, and Mito + ™ Serum Extender (Cat. no. 355006; Becton Dickinson Immunocytometry Systems, San Jose, CA). PHA was prepared in RPMI-1640 media at a concentration of 1 mg/mL and was then further diluted with complete media to the optimal working concentration (6.25 μg/mL). A 100 μL aliquot of the diluted blood was dispensed into each of triplicate wells of a 96-well flat-bottom microtiter plate. To each well, 100 μL of the appropriate mitogen concentration was added. Control wells received complete media instead of mitogen. After 72 h incubation at 37°C and 5% CO2, the cells were pulsed with 1 μCi of [3H]-thymidine (New England Nuclear, Boston, MA) prepared with RPMI-1640. After pulsing, cells were incubated for an additional 4 h before harvesting. The radionucleotide incorporation was assessed using a Wallac 1409 RackBeta liquid scintillation counter (LKB Wallac, Inc., Gaithersburg, MD) with the results expressed as experimental minus control counts per minute (cpm).

### Statistical analysis

Data were subjected to analysis of variance according to the general linear model (GLM) procedure of the Statistical Analysis System software package version 6.11 (SAS Institute, Cary, NC, USA). Repeated measures analysis of variance with time and treatment as the within-subject factor was used to analyze blood count, salivary IgA and PHA-stimulated lymphocyte proliferation over time using the model MIXED-type TOEP of SAS, and LSMEANS follow-up test was used for comparisons of means. A two-tailed P-value of < 0.05 was considered significant.

## Results

**Figure 1:**
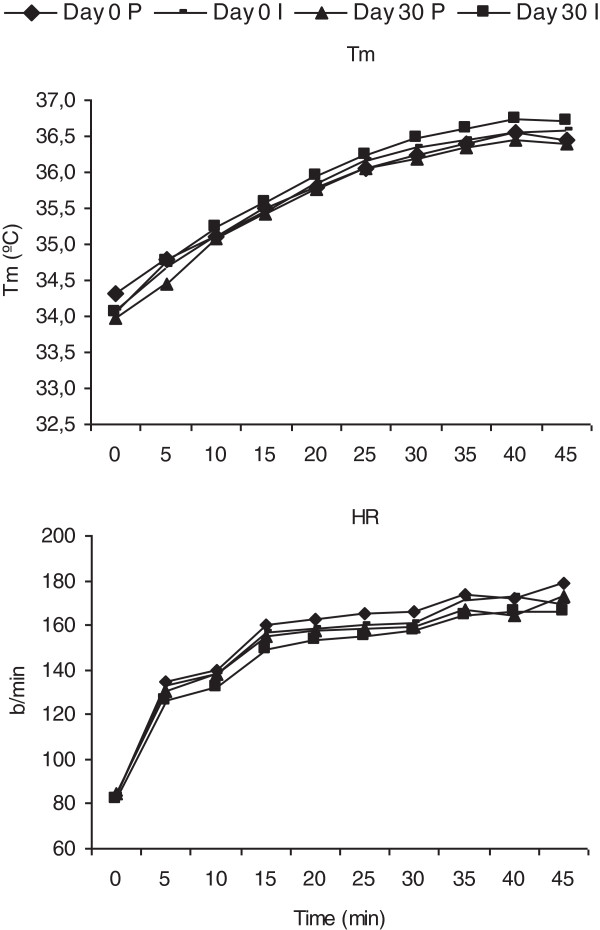
**Mean temperature and heart rate during exercise before and after 30 days of supplementation.** Values are means ± SE (n = 10). P = Placebo; I = Inmunactive. Tm = Mean temperature; HR = Heart Rate.

Basal subject characteristics and performance data for the 20 subjects are summarized in Table 1. No significant differences were found between groups for age, body composition, or maximal performance measures. The mean temperature and% of humidity in the climatic chamber were -2.5 ± 1.4°C and 67 ± 7.3% for day 0, and -2.3 ± 2.8°C and 60.72 ± 5.0% for day 30. The exercise test had an average duration of 47.3 ± 5.3 minutes. T_sk_, T_c_ and T_m_ increased during the exercise test and reached a physiological steady state. Figure 1 and Table 2 shows that thermoregulation mechanisms were not compromised during the exercise tests and weight loss was less than 1% (0.77% and 0.71%).

**Table 1:** Subjects characteristics and performance at baseline

**Variable**	**Inmunactive**	**Placebo**
Age (yr)	22.8 ± 5	21.9 ± 3
Body mass (kg)	70.7 ± 4.7	75.8 ± 3.25
Body composition (% fat)	11.6 ± 3.5	9.5 ±3.8
VO_2max_ (mL · kg^-1^ · min^-1^)	45.8 ± 5.3	47.3 ± 7.0

VO_2max_ = maximal oxygen uptake. Values are presented as means ± SE (n = 10).

No differences between groups were detected.

**Table 2:** Maximal physiological and performance values on exercise tests before and after 30 days of supplementation

**Variable**	**Day 0 Inmunactive**	**Placebo**	**Day 30 Inmunactive**	**Placebo**
Skin temperature (°C)	29.1 ± 0.4	29.4 ± 0.3	30.4 ± 0.6	29.4 ± 0.7
Core temperature (°C)	38.5 ± 0.1	38.5 ± 0.1	38.5 ± 0.1	38.2 ± 0.1
Heart rate (bpm)	176 ± 3	179 ± 3	171 ± 4	171 ± 3
Lactate (mM)	10.1 ± 0.9	9.9 ± 1.1	8.7 ± 0.6	7.7 ± 1.4
Borg scale	18.8 ± 0.1	18.6 ± 0.2	18 ± 0.4	17.9 ± 0.5

Values are means ± SE (n = 10). No significant differences across time within a group treatment or between groups at specified time point were detected.

On day 0 the average HR_max_ of all the participants reached 177 ± 2 bpm corresponding to 96% of the HR_max_ determined in the maximal incremental test (Table 2). On day 30 the average HR_max_ showed a decreasing trend (171 ± 2 bpm; P = 0.06). Maximal lactate concentration tended to be lower (P = 0.09) and maximal RPE was significantly lower (P = 0.03) on day 30 in comparison with day 0. No differences between treatment groups were detected in performance parameters, except for RPE recorded during the exercise test on day 30. Thus values were lower for I compared to P group at 10 min (9.9 ± 0.5 vs. 11.2 ± 0.7; P = 0.0496), 20 min (13.2 ± 0.6 vs. 14.7 ± 0.3; P = 0.0238) and 30 min (15.2 ± 0.6 vs. 16.6 ± 0.4; P = 0.0347) but not at the end of the test (40 min; 17.2 ± 0.5 vs. 17.6 ± 0.4; P = 0.4582). The maximum Borg value was not different between groups (Table 2), because all the participants reached exhaustion before finishing the test.

Seven-day food records before the exercise test revealed no significant group differences in energy or macronutrient intake. Energy intake was 2195 ± 527 Kcal, containing 247 ± 66 g of carbohydrate, 88.4 ± 24.1 g of fat and 100 ± 25 g of protein for all subject combined. Similarly, no changes in food intake were recorded thorough the study period.

To avoid the influence of possible changes in plasma volume caused by exercise, CBC was adjusted following the methodology proposed by Dill and Costill [31]. There was a significant effect of time during the exercise test (basal, 30 min, 150 min) for total leukocyte, neutrophil an lymphocyte counts (P < 0.05) (Table 3), namely an increase in total leukocyte and neutrophil counts at 150 min after exercise and a decrease in lymphocyte counts 30 and 150 min after exercise. However no differences between groups or across exercise tests (day 0 and day 30) were detected in the pattern of response except for the lymphocyte counts. Thus on day 30 the I group, supplemented with nucleotides, did experience a decrease in lymphocyte counts at 30 min compared to the basal values but a total recovery was registered at 150 min, while the placebo group stayed low and was significantly reduced compared with the I group (P = 0.0028).

**Table 3:** Blood count during exercise tests before and after 30 days of supplementation

**Variable**	**Day 0 Inmunactive**	**Placebo**	**Day 30 Inmunactive**	**Placebo**
Total leukocytes (10^9^ · L^-1^)				
Basal	6.38 ± 0.53^b^	6.10 ± 0.47^b^	7.00 ± 0.71^b^	5.25 ± 0.44^b^
30 min	6.34 ± 0.76^b^	6.72 ± 0.93^b^	6.83 ± 0.74^b^	5.66 ± 0.72^b^
150 min	10.45 ± 1.19^a^	9.86 ± 1.03^a^	10.36 ± 0.86^a^	8.32 ± 0.96ª
Neutrophils (10^9^ · L^-1^)				
Basal	3.69 ± 0.35^c^	3.27 ± 0.41^b^	4.06 ± 0.43^b^	2.98 ± 0.38^b^
30 min	4.30 ± 0.70^b^	4.65 ± 0.87^b^	4.30 ± 0.54^b^	3.83 ± 0.70^b^
150 min	8.06 ± 0.89^a^	7.80 ± 1.01^a^	7.27 ± 0.59^a^	7.17 ± 1.05ª
Lymphocytes (10^9^ · L^-1^)				
Basal	2.03 ± 0.14ª	2.03 ± 0.13ª	2.12 ± 0.22ª	1.73 ± 0.12ª
30 min	1.37 ± 0.09^b^	1.39 ± 0.12^b^	1.77 ± 0.17^b^	1.44 ± 0.09^b^
150 min	1.68 ± 0.11^b^	1.43 ± 0.11^b^	2.27 ± 0.37ª*	1.50 ± 0.07^ab^

Values are means ± SE (n = 10). Different superscripts indicate significant differences across time within a group treatment. An asterisk indicates significant differences between groups at specified time point (P < 0.05).

There was no effect of time (basal or 150 min), exercise test (day 0 or day 30) or treatment group on salivary IgA concentration (P > 0.05) (Table 4). Similarly, there was no significant effect of exercise on the lymphoproliferative response, although an almost significant decrease was observed in the I group at baseline, i.e. prior to treatment (P < 0.06, Table 4). This resulted in a lower lymphocyte proliferation in the treated group. Despite this apparently higher susceptibility to exercise-evoked depression in the lymphoproliferative response, the I group exhibited a significantly higher proliferation after the 30 day supplementation period, indicating a full reversal of this tendency. In keeping with this, the statistical analysis showed a significant day*group interaction (P = 0.0045).

**Table 4:** Salivary IgA and PHA-Stimulated lymphocyte proliferation during exercise tests before and after 30 days of supplementation

**Variable**	**Day 0 Inmunactive**	**Placebo**	**Day 30 Inmunactive**	**Placebo**
Salivary IgA (mg · L^-1^)				
Basal	1.87 ± 0.38	2.59 ± 1.16	2.32 ± 0.96	2.31 ± 0.61
150 min	2.43 ± 1.06	2.13 ± 0.70	1.91 ± 0.54	1.35 ± 0.45
PHA-Stimulated lymphocyte proliferation (cpm · 1000^-1^)				
Basal	29.3 ± 3.5	35.5 ± 4.4	29.1 ± 2.1	25.9 ± 3.9
24 h	21.4 ± 3.6	35.9 ± 53.8*	34.5 ± 5.4	20.6 ± 5.1*

Values are means ± SE (n = 10). An asterisk indicates significant differences between groups at specified time point (P < 0.05).

## Discussion

Scientific evidence from placebo-controlled trials of nutritional compounds having a positive enhancing effect on the immune function in the healthy population is scarce [32]. High-intensity exercise has been classically associated to immune disturbances in healthy individuals [2] and thus could be considered as a model to study the efficacy of nutritional interventions in populations during periods of immune suppression [33]. Exposure to cold environments has been claimed to elicit a stress response impacting immune cell function [10], but evidences from controlled studies are also scarce [13]. Research on the potential for dietary nucleotides to enhance the human immune response is wide but human trials are mainly restricted to critically ill patients [34] and to supplementation of infant formula [35]. To our knowledge, this is the first controlled study in which the efficacy of nucleotide supplementation has been evaluated in healthy individuals under multiple stressors such as strenuous exercise and cold environment.

The exercise protocol was designed to elicit an immune disturbance according to previously published data [4,36]. Subjects were instructed to perform a controlled physical work corresponding to 90% of the VO_2max_ for more than 20 minutes, in an exercise bout of more than 45 minutes in total. The described workload led to exhaustion as demonstrated by the maximum heart rate, lactate concentration and Borg values. On the second exercise test, Borg values were lower and HR_max_ and lactate concentration tended to be lower than in the previous exercise test, probably due to the effect of the training during the month of the trial. Levels of salivary IgA were unaffected by the exercise. Although falls in saliva IgA can occur during intense exercise [37-39], levels are generally unchanged with exercise lasting less than 1 h [40] and also not affected by environmental temperature [41-43], as observed in the present trial. Nevertheless, in the present trial the workload in first and second exercise tests was enough to elicit a profound leukocytosis and neutrophilia. Marked changes in blood leukocyte counts resulting from a single bout of high intensity exercise are well known and are due largely to the movement of neutrophils from the marginal pool to the circulating pool as a result of muscular action [44]. It is documented that neutrophilia depends of exercise intensity and duration [7] and also of body temperature attained during exercise [45]. Acute exercise results in a rapid increase in blood neutrophil counts likely due to demargination caused by shear stress and catecholamines [46], which is followed by a delayed neutrophilia attributed to cortisol-induced release of neutrophils from the bone marrow [46]. An increase in blood neutrophil numbers does not imply better neutrophil function, because neutrophils released as a result of acute exercise are relatively immature and consequently their degranulation and oxidative burst in response to bacterial stimulation may be reduced for many hours after the exercise bout [47-49]. Acute exercise elicits characteristic transient biphasic changes in the numbers of circulating lymphocytes. Typically, a lymphocytosis is observed immediately after exercise, with numbers of cells falling below pre-exercise levels during the early stages of recovery [50]. Results obtained in this study are in total agreement with this pattern of response, with significant decreases in lymphocyte numbers detected at 30 and 150 min after exercise, except for the group supplemented with nucleotides in which a total recovery on the number of lymphocytes was detected at 150 min. Although it has been shown that dietary nucleotides stimulates the maturation of immune cells [17,51], the rapid recovery in lymphocyte counts registered between 30 and 150 min after the exercise test, suggest a redistribution from other cell compartments.

There is considerable evidence demonstrating that exogenous nucleotides increase the proliferative response to T cell-dependent mitogens (PHA, ConA and PWM) [14,17]. In the present study, significant differences in lymphocyte proliferation have been detected between treatment groups at 24 h after exercise. On the initial exercise test, lymphoproliferative activity was higher in the placebo group (P < 0.05), while after supplementation it was higher in the nucleotide group (P < 0.05). Interpretation of the data is hampered by the fact that values are different in the baseline test. This was probably due to the reduced sample size (10 athletes per group) and the randomized nature of the study, which resulted by happenstance (since this result is prior to intervention) in an almost significant effect of exercise in the I group. This may be interpreted to indicate a higher susceptibility of this group to depressed lymphocyte proliferation in the face of intense physical activity. This in turn would be expected to dampen, or hide, a putative effect of the nucleotide supplement in this regard. Despite this obstacle, Inmunactive effectively augmented the lymphoproliferative response in the treated group, suggesting that this is a consistent effect. Previous studies using standard lymphocyte proliferation assays have reported significant reductions in T-lymphocyte responses to mitogen after medium- and long-duration intense exercise [52], which have been suggested to explain the observed high incidence of infections in elite athletes [53,54]. These reductions of proliferative responses have been attributed to an increase in cell death of both CD4 and CD8 T lymphocytes, rather than to decrease in mitosis rate [55]. The molecular mechanisms by which dietary nucleotides exert their effects are largely unknown, but recent findings have demonstrated that they affect the expression and activity of several transcriptional factors involved in cell growth, differentiation and apoptosis [56]. Specifically exogenous nucleotides have shown to reduce the expression and activity of the glucocorticoid receptor NR3C1, the upstream stimulatory factor USF1, NF-κB and the tumor protein p53. TP53 responds to diverse cellular stresses to regulate target genes that induce cell arrest, apoptosis and senescence [57].

## Conclusion

Our results suggest that exogenous nucleotides may have a protective effect on the on the markers immune response of athletes after strenuous exercise. According to the recent findings, it could be hypothesized that this protection could be mediated by a preventive effect against apoptosis induced by different stress stimuli. However further studies are required to elucidate the mechanisms of action of dietary nucleotides, as well as to evaluate their potential in prevention of immune disturbances.

## Competing interests

Financial support for this work was provided by Bioiberica S.A. (Palafolls, Spain).

## Authors’ contributions

JR and VP were the study coordinators and were involved in research design, data collection and analysis, as well as manuscript preparation. DM and CC were involved in research design, analysis and manuscript preparation. JAT, AP and FD assisted in research design and analysis. All authors read and approved the final manuscript.
